# Complete Genome Sequence of Bacillus subtilis Strain WB800N, an Extracellular Protease-Deficient Derivative of Strain 168

**DOI:** 10.1128/MRA.01380-18

**Published:** 2018-11-08

**Authors:** Haeyoung Jeong, Da-Eun Jeong, Seung-Hwan Park, Seong Joo Kim, Soo-Keun Choi

**Affiliations:** a Infectious Disease Research Center, Korea Research Institute of Bioscience and Biotechnology (KRIBB), Yuseong-gu, Daejeon, Republic of Korea; b Department of Biosystems and Bioengineering, KRIBB School of Biotechnology, Korea University of Science and Technology (UST), Yuseong-gu, Daejeon, Republic of Korea; c The 4th R&D Institute, Agency for Defense Development (ADD), Daejeon, Republic of Korea; Broad Institute of MIT and Harvard University

## Abstract

Bacillus subtilis WB800N is a genetically engineered variant of B. subtilis 168, such that all extracellular proteases are disrupted, which enables WB800N to be widely used for the expression of secretory proteins. Here, we report the 4.2-Mb complete genome sequence of WB800N and present all of the disrupted gene structure.

## ANNOUNCEMENT

Bacillus subtilis has many advantages as a protein expression host, such as its generally recognized as safe (GRAS) status, easy genetic manipulation, well-known large-scale fermentation process, and protein secretion into media. However, the expression of recombinant secretory proteins in B. subtilis has often been unsuccessful due to the degradation of secreted proteins by extracellular proteases (1). B. subtilis has eight extracellular proteases, known as NprE, AprE, Epr, Bpr, Mpr, NprB, Vpr, and WprA. To increase the stability of secreted proteins, the eight-extracellular-protease-deficient mutant WB800N was constructed (2) and is commercially available. However, since it was constructed over a long period of time, it is not easy to follow the construction process in order to know the accurate genetic structure of the extracellular protease genes on the genome. Furthermore, the insertion locus of the kanamycin resistance gene is unclear. To clarify this uncertainty, we determined the complete genome sequence of B. subtilis WB800N for further strain improvement.

WB800N cells (catalog no. PBS022) were purchased from MoBiTec (Göttingen, Germany) and were grown aerobically in Luria-Bertani (LB) medium at 37°C. Genomic DNA was extracted using a Wizard genomic DNA purification kit from Promega (Wisconsin, USA) according to the manufacturer’s instructions. Library construction and genome sequencing were carried out on a PacBio RS II platform at Chun Lab (Seoul, Republic of Korea) using P6-C4 chemistry. Using the RS_HGAP_Assembly.3 protocol in SMRT Analysis v2.3 (https://www.pacb.com/products-and-services/analytical-software/smrt-analysis/), 73,369 reads totaling 361.44 Mb (81.3× genome coverage and an *N*_50_ read length of 7,290 bp) were assembled into two contigs of 4.23 Mb and 1.88 kb. The smaller contig was discarded because it was found to be the PacBio internal control DNA. The chromosomal sequence, corrected through two consecutive rounds of the RS_Resequencing.1 protocol in SMRT Analysis, was circularized using Circlator (3). The final sequence consists of a chromosome of 4,214,174 bp with a G+C content of 43.5%.

**FIG 1:**
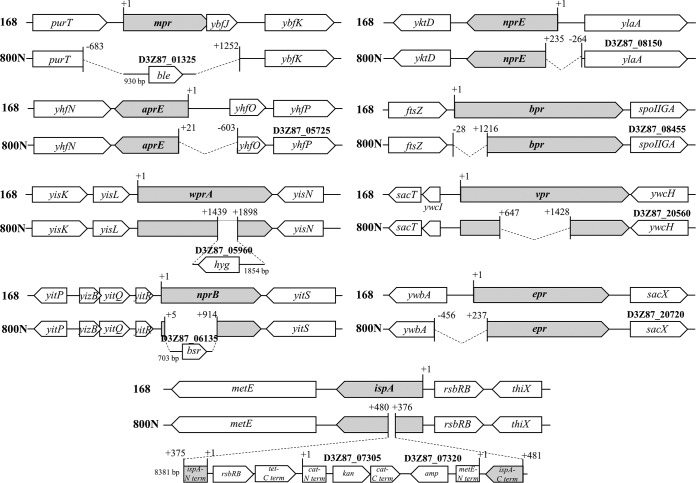
Comparison of protease gene structure in the genome between B. subtilis strains 168 and WB800N. The numbers indicate the distance from the translation start sites (+1). Resistance gene abbreviations: *ble*, bleomycin; *bsr*, blasticidin; *hyg*, hygromycin; *tet*, tetracycline; *cat*, chloramphenicol; *kan*, kanamycin; *amp*, ampicillin. Bold numbers represent the locus tag for each gene within the genome sequence.

Genome annotation was carried out using NCBI’s Prokaryotic Genome Annotation Pipeline (PGAP) v4.6 (4). Compared to the genome sequence of B. subtilis strain 168 (GenBank accession no. NC_000964.3) using Cross_match (http://www.phrap.org/), all alignment blocks were nearly identical with each other (0 to 0.01% nucleotide difference), and they were placed collinearly along the chromosome. Unaligned regions at the boundaries of each block are due to the genetic manipulations in the WB800N strain. Compared to strain 168, the gene structure changes of WB800N are as follows. The *nprE*, *aprE*, *bpr*, *vpr*, and *epr* genes were partially deleted without insertion of the antibiotic resistance gene, while *wprA* and *nprB* were disrupted by the hygromycin resistance gene and the blasticidin resistance gene, respectively. The entire *mpr* gene was deleted and replaced by the bleomycin resistance gene. In the middle of the *ispA* gene, a large DNA fragment containing an *rsbRB*, a C-terminal fragment of a tetracycline resistance gene, a separated chloramphenicol resistance gene, a kanamycin resistance gene, an ampicillin resistance gene, and an N-terminal fragment of *metE* were inserted (Fig. 1). The genome information will be useful for further improvement of the strain.

### Data availability.

This genome sequencing project has been deposited in DDBJ/ENA/GenBank under the accession no. CP032310. The version described in this paper is the first version (CP032310.1). Raw sequencing reads are available in NCBI under BioProject accession no. PRJNA490410.
